# 5-Bromo-2-hy­droxy­benzaldehyde 4-ethyl­thio­semicarbazone

**DOI:** 10.1107/S1600536813008787

**Published:** 2013-04-20

**Authors:** Zhaoyang Li, Osamu Sato

**Affiliations:** a Institute for Materials Chemistry and Engineering, Kyushu University, 6-1 Kasuga-koen, Kasuga, Fukuoka 816-8580, Japan

## Abstract

In the title Schiff base compound, C_10_H_12_BrN_3_OS, the C—N—N—C torsion angle is 172.07 (11)°. An intra­molecular hydrogen bond exists between the hy­droxy H atom and the azomethine N atom. In the crystal, pairs of hydrogen bonds involving the imino H atom and the S atom give rise to supra­molecular dimers.

## Related literature

For the isostructural compound 5-chloro-2-hy­droxy­benz­alde­hyde 4-ethyl­thio­semicarbazone, see: Lo *et al.* (2011 ▶)
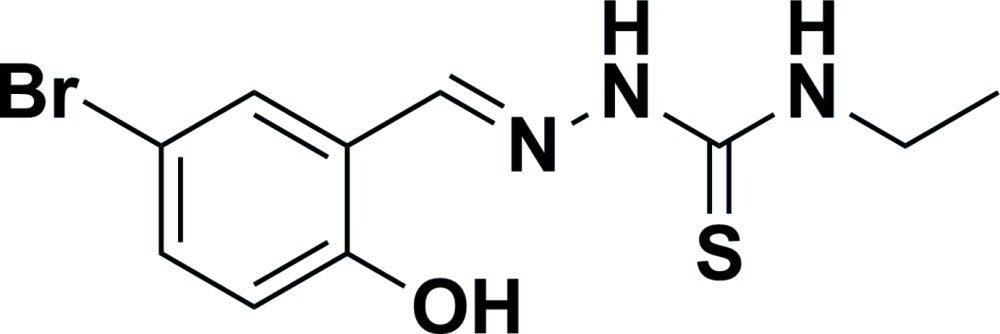


## Experimental

### 

#### Crystal data

C_10_H_12_BrN_3_OS
*M* *_r_* = 302.20
Monoclinic, 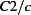

*a* = 22.040 (4) Å
*b* = 11.844 (2) Å
*c* = 9.5102 (19) Å
β = 101.69 (3)°
*V* = 2431.1 (8) Å^3^
*Z* = 8
Mo *K*α radiation
μ = 3.54 mm^−1^
*T* = 123 K
0.20 × 0.10 × 0.05 mm

#### Data collection

Rigaku Saturn70 diffractometer
Absorption correction: multi-scan (*CrystalClear*; Rigaku, 2008 ▶) *T*_min_ = 0.661, *T*_max_ = 0.838
4201 measured reflections
2331 independent reflections
1760 reflections with *I* > 2σ(*I*)
*R*_int_ = 0.032

#### Refinement

*R*[*F* ^2^ > 2σ(*F* ^2^)] = 0.042
*wR*(*F* ^2^) = 0.114
*S* = 0.95
2331 reflections
155 parameters
3 restraints
H atoms treated by a mixture of independent and constrained refinement
Δρ_max_ = 0.70 e Å^−3^
Δρ_min_ = −1.01 e Å^−3^

### 

Data collection: *CrystalClear* (Rigaku, 2008 ▶); cell refinement: *CrystalClear*; data reduction: *CrystalClear*; program(s) used to solve structure: *SHELXS97* (Sheldrick, 2008 ▶); program(s) used to refine structure: *SHELXL97* (Sheldrick, 2008 ▶); molecular graphics: *SHELXTL* (Sheldrick, 2008 ▶); software used to prepare material for publication: *publCIF* (Westrip, 2010 ▶).

## Supplementary Material

Crystal structure: contains datablock(s) I, global. DOI: 10.1107/S1600536813008787/ng5322sup1.cif

Structure factors: contains datablock(s) I. DOI: 10.1107/S1600536813008787/ng5322Isup2.hkl

Supplementary material file. DOI: 10.1107/S1600536813008787/ng5322Isup3.cml

Additional supplementary materials:  crystallographic information; 3D view; checkCIF report

## Figures and Tables

**Table 1:** Hydrogen-bond geometry (Å, °)

*D*—H⋯*A*	*D*—H	H⋯*A*	*D*⋯*A*	*D*—H⋯*A*
O1—H1*A*⋯N1	0.84 (3)	2.00 (2)	2.674 (3)	137 (3)
N2—H2*A*⋯S1^i^	0.88 (3)	2.47 (3)	3.316 (3)	161 (2)
N3—H3*A*⋯S1^ii^	0.87 (3)	2.75 (3)	3.510 (3)	146 (3)

Symmetry codes: (i) 
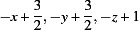
; (ii) 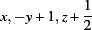.

**Fig. 1.**
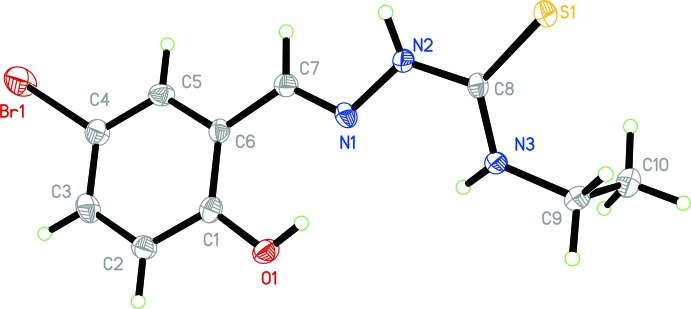
Displacement ellipsoid plot (50% probability level) of the title compound, with atom numbering of structurally unique non-H atoms and the H atoms.

**Fig. 2.**
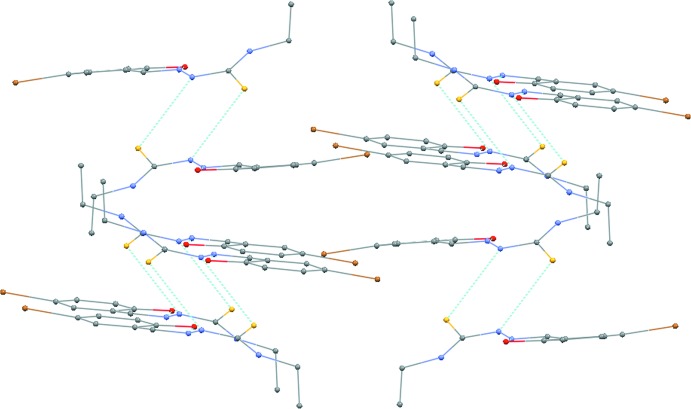
The packing diagram of the title compound, with H atoms omitted for clarity. Hydrogen bonds are shown as dashed lines.

## supplementary crystallographic information

### Comment

A Schiff ligand was synthesized through one-pot reaction with high yield using 5-bromo-2-hydroxybenzaldehyde and 4-ethyl-3-thiosemicarbazide (Fig. 1). The title compound can be used as tridentate chelating ligand to construct spin-crossover complexes. Isostructural 5-chloro-2-hydroxybenzaldehyde-4-ethylthiosemicarbazone was reported previously (Lo *et al.*, 2011).

In the title compound, a strong intramolecular hydrogen bond O—H···N is observed. An intermolecular N—H···S hydrogen bond connects two molecules into a supramolecular dimer as shown in Figure 2.

### Experimental

5-Bromo-2-hydroxybenzaldehyde (4.02 g, 20 mmol) in 50 ml ethanol and 4-ethyl-3-thiosemicarbazide (2.38 g, 20 mmol) were reacted for 6 h at 350 K. Slow evaporation of the yellow solution gave large colorless crystals.

### Refinement

Carbon-bound H-atoms were placed in calculated positions (C—H 0.95, 0.98 and 0.99 Å) and were included in the refinement in the riding model approximation, with *U*_iso_(H) =1.5U_eq_(C) for methyl H atoms and 1.2U_eq_(C) for the others. The hydroxy and amino H atoms were located in a difference Fourier map, and were refined with distance restraints of O—H 0.85±0.01 and N—H 0.88±0.01 Å; with *U*_iso_(H) =1.2U_eq_(N and O).

### Crystal data

**Table:**

C_10_H_12_BrN_3_OS	*F*(000) = 1216
*M**_r_* = 302.20	*D*_x_ = 1.651 Mg m^−^^3^
Monoclinic, *C*2/*c*	Mo *K*α radiation, λ = 0.710747 Å
Hall symbol: -C 2yc	Cell parameters from 3650 reflections
*a* = 22.040 (4) Å	θ = 3.1–27.5°
*b* = 11.844 (2) Å	µ = 3.54 mm^−^^1^
*c* = 9.5102 (19) Å	*T* = 123 K
β = 101.69 (3)°	Block, colourless
*V* = 2431.1 (8) Å^3^	0.20 × 0.10 × 0.05 mm
*Z* = 8	

### Data collection

**Table:**

Rigaku Saturn70 diffractometer	2331 independent reflections
Radiation source: Rotating Anode	1760 reflections with *I* > 2σ(*I*)
Confocal monochromator	*R*_int_ = 0.032
Detector resolution: 28.5714 pixels mm^-1^	θ_max_ = 26.0°, θ_min_ = 3.1°
dtprofit.ref scans	*h* = −27→20
Absorption correction: multi-scan (*CrystalClear*; Rigaku, 2008)	*k* = −9→14
*T*_min_ = 0.661, *T*_max_ = 0.838	*l* = −11→10
4201 measured reflections	

### Refinement

**Table:**

Refinement on *F*^2^	Primary atom site location: structure-invariant direct methods
Least-squares matrix: full	Secondary atom site location: difference Fourier map
*R*[*F*^2^ > 2σ(*F*^2^)] = 0.042	Hydrogen site location: inferred from neighbouring sites
*wR*(*F*^2^) = 0.114	H atoms treated by a mixture of independent and constrained refinement
*S* = 0.95	*w* = 1/[σ^2^(*F*_o_^2^) + (0.0752*P*)^2^] where *P* = (*F*_o_^2^ + 2*F*_c_^2^)/3
2331 reflections	(Δ/σ)_max_ = 0.001
155 parameters	Δρ_max_ = 0.70 e Å^−^^3^
3 restraints	Δρ_min_ = −1.01 e Å^−^^3^

### Special details

**Table:**

Geometry. All e.s.d.'s (except the e.s.d. in the dihedral angle between two l.s. planes) are estimated using the full covariance matrix. The cell e.s.d.'s are taken into account individually in the estimation of e.s.d.'s in distances, angles and torsion angles; correlations between e.s.d.'s in cell parameters are only used when they are defined by crystal symmetry. An approximate (isotropic) treatment of cell e.s.d.'s is used for estimating e.s.d.'s involving l.s. planes.
Refinement. Refinement of *F*^2^ against ALL reflections. The weighted *R*-factor *wR* and goodness of fit *S* are based on *F*^2^, conventional *R*-factors *R* are based on *F*, with *F* set to zero for negative *F*^2^. The threshold expression of *F*^2^ > σ(*F*^2^) is used only for calculating *R*-factors(gt) *etc*. and is not relevant to the choice of reflections for refinement. *R*-factors based on *F*^2^ are statistically about twice as large as those based on *F*, and *R*- factors based on ALL data will be even larger.

### Fractional atomic coordinates and isotropic or equivalent isotropic displacement parameters (Å^2^)

**Table:**

	*x*	*y*	*z*	*U*_iso_*/*U*_eq_	
Br1	0.484097 (17)	0.67444 (4)	1.01744 (4)	0.04059 (19)	
C1	0.69767 (15)	0.6276 (2)	1.0597 (3)	0.0180 (7)	
C2	0.67216 (16)	0.6364 (3)	1.1818 (3)	0.0197 (7)	
H2	0.6985	0.6352	1.2742	0.024*	
C3	0.60893 (17)	0.6470 (3)	1.1699 (4)	0.0227 (7)	
H3	0.5919	0.6537	1.2536	0.027*	
C4	0.57021 (16)	0.6476 (3)	1.0343 (4)	0.0222 (7)	
C5	0.59471 (16)	0.6361 (3)	0.9125 (3)	0.0198 (7)	
H5	0.5678	0.6343	0.8208	0.024*	
C6	0.65841 (15)	0.6271 (3)	0.9228 (3)	0.0164 (7)	
C7	0.68243 (15)	0.6269 (3)	0.7906 (3)	0.0179 (7)	
H7	0.6542	0.6340	0.7012	0.021*	
C8	0.81461 (14)	0.6124 (2)	0.6421 (3)	0.0150 (6)	
C9	0.91521 (15)	0.5238 (3)	0.7381 (3)	0.0216 (7)	
H9A	0.9408	0.5182	0.8363	0.026*	
H9B	0.9339	0.5821	0.6855	0.026*	
C10	0.91576 (17)	0.4111 (3)	0.6625 (4)	0.0270 (8)	
H10A	0.9008	0.3519	0.7190	0.040*	
H10B	0.9581	0.3935	0.6524	0.040*	
H10C	0.8887	0.4152	0.5672	0.040*	
H1A	0.7730 (17)	0.634 (3)	1.002 (2)	0.032*	
H2A	0.7325 (16)	0.680 (2)	0.600 (3)	0.032*	
H3A	0.8346 (17)	0.525 (3)	0.810 (3)	0.032*	
N1	0.74029 (12)	0.6174 (2)	0.7914 (3)	0.0169 (6)	
N2	0.75628 (13)	0.6333 (2)	0.6594 (3)	0.0177 (6)	
N3	0.85244 (13)	0.5580 (2)	0.7468 (3)	0.0172 (6)	
O1	0.75977 (11)	0.62315 (19)	1.0781 (2)	0.0207 (5)	
S1	0.83506 (4)	0.65794 (7)	0.48834 (9)	0.0201 (2)	

### Atomic displacement parameters (Å^2^)

**Table:**

	*U*^11^	*U*^22^	*U*^33^	*U*^12^	*U*^13^	*U*^23^
Br1	0.0165 (2)	0.0815 (4)	0.0261 (2)	0.00156 (19)	0.00976 (16)	0.00158 (19)
C1	0.0186 (18)	0.0130 (14)	0.0228 (17)	0.0006 (13)	0.0052 (14)	−0.0009 (13)
C2	0.0227 (19)	0.0187 (15)	0.0172 (16)	0.0008 (13)	0.0031 (14)	0.0002 (13)
C3	0.026 (2)	0.0234 (16)	0.0223 (16)	−0.0019 (14)	0.0134 (15)	0.0023 (14)
C4	0.0145 (18)	0.0314 (18)	0.0221 (17)	−0.0023 (14)	0.0066 (14)	−0.0001 (14)
C5	0.0163 (17)	0.0239 (16)	0.0180 (16)	−0.0019 (13)	0.0010 (13)	0.0013 (13)
C6	0.0173 (17)	0.0151 (14)	0.0180 (16)	0.0018 (13)	0.0065 (13)	0.0018 (13)
C7	0.0176 (17)	0.0187 (15)	0.0172 (15)	0.0003 (13)	0.0032 (13)	0.0011 (13)
C8	0.0166 (17)	0.0131 (14)	0.0162 (15)	0.0007 (12)	0.0056 (13)	−0.0023 (13)
C9	0.0152 (17)	0.0291 (17)	0.0198 (16)	0.0029 (14)	0.0017 (13)	0.0026 (14)
C10	0.021 (2)	0.033 (2)	0.0274 (18)	0.0060 (15)	0.0064 (15)	−0.0024 (15)
N1	0.0195 (15)	0.0169 (12)	0.0158 (13)	−0.0005 (11)	0.0073 (11)	0.0006 (11)
N2	0.0166 (15)	0.0213 (13)	0.0164 (13)	0.0046 (11)	0.0060 (11)	0.0034 (11)
N3	0.0145 (14)	0.0227 (14)	0.0145 (13)	0.0024 (11)	0.0036 (11)	0.0030 (11)
O1	0.0153 (13)	0.0259 (12)	0.0205 (12)	0.0017 (10)	0.0031 (10)	0.0040 (10)
S1	0.0192 (5)	0.0253 (4)	0.0175 (4)	0.0038 (3)	0.0079 (3)	0.0032 (3)

### Geometric parameters (Å, º)

**Table:**

Br1—C4	1.899 (4)	C8—N3	1.329 (4)
C1—O1	1.345 (4)	C8—N2	1.351 (4)
C1—C2	1.393 (5)	C8—S1	1.703 (3)
C1—C6	1.410 (5)	C9—N3	1.460 (4)
C2—C3	1.381 (5)	C9—C10	1.517 (5)
C2—H2	0.9500	C9—H9A	0.9900
C3—C4	1.395 (5)	C9—H9B	0.9900
C3—H3	0.9500	C10—H10A	0.9800
C4—C5	1.380 (5)	C10—H10B	0.9800
C5—C6	1.391 (4)	C10—H10C	0.9800
C5—H5	0.9500	N1—N2	1.384 (3)
C6—C7	1.460 (4)	N2—H2A	0.879 (10)
C7—N1	1.278 (4)	N3—H3A	0.876 (10)
C7—H7	0.9500	O1—H1A	0.846 (10)
			
O1—C1—C2	117.8 (3)	N3—C8—S1	124.2 (2)
O1—C1—C6	122.5 (3)	N2—C8—S1	118.0 (2)
C2—C1—C6	119.6 (3)	N3—C9—C10	111.7 (3)
C3—C2—C1	120.6 (3)	N3—C9—H9A	109.3
C3—C2—H2	119.7	C10—C9—H9A	109.3
C1—C2—H2	119.7	N3—C9—H9B	109.3
C2—C3—C4	119.7 (3)	C10—C9—H9B	109.3
C2—C3—H3	120.2	H9A—C9—H9B	107.9
C4—C3—H3	120.2	C9—C10—H10A	109.5
C5—C4—C3	120.4 (3)	C9—C10—H10B	109.5
C5—C4—Br1	120.0 (3)	H10A—C10—H10B	109.5
C3—C4—Br1	119.5 (3)	C9—C10—H10C	109.5
C4—C5—C6	120.6 (3)	H10A—C10—H10C	109.5
C4—C5—H5	119.7	H10B—C10—H10C	109.5
C6—C5—H5	119.7	C7—N1—N2	114.8 (3)
C5—C6—C1	119.1 (3)	C8—N2—N1	120.6 (3)
C5—C6—C7	118.4 (3)	C8—N2—H2A	120 (3)
C1—C6—C7	122.2 (3)	N1—N2—H2A	116 (3)
N1—C7—C6	122.0 (3)	C8—N3—C9	123.4 (3)
N1—C7—H7	119.0	C8—N3—H3A	115 (3)
C6—C7—H7	119.0	C9—N3—H3A	119 (3)
N3—C8—N2	117.8 (3)	C1—O1—H1A	114 (3)

### Hydrogen-bond geometry (Å, º)

**Table:**

*D*—H···*A*	*D*—H	H···*A*	*D*···*A*	*D*—H···*A*
O1—H1*A*···N1	0.84 (3)	2.00 (2)	2.674 (3)	137 (3)
N2—H2*A*···S1^i^	0.88 (3)	2.47 (3)	3.316 (3)	161 (2)
N3—H3*A*···S1^ii^	0.87 (3)	2.75 (3)	3.510 (3)	146 (3)

Symmetry codes: (i) −*x*+3/2, −*y*+3/2, −*z*+1; (ii) *x*, −*y*+1, *z*+1/2.
